# The Role of C-Reactive Protein as a Biomarker for Postoperative Delirium Following Cardiac and Neurosurgical Surgery: A Retrospective Analysis

**DOI:** 10.3390/jcm14228252

**Published:** 2025-11-20

**Authors:** Mateusz Szczupak, Jacek Kobak, Anna Ingielewicz, Jakub Wiśniewski, Sabina Krupa-Nurcek

**Affiliations:** 1 Department of Anesthesiology and Intensive Care, Copernicus Hospital in Gdansk, 80-803 Gdansk, Poland; szczupak.mateusz@icloud.com; 2 Department of Otolaryngology, Faculty of Medicine, Medical University of Gdansk, 80-210 Gdansk, Poland; 3 Department of Emergency Medicine, Faculty of Health Science, Medical University of Gdańsk, 80-210 Gdansk, Poland; anna.ingielewicz@gumed.edu.pl; 4 Department of Neurosurgery, Copernicus Hospital in Gdansk, 80-803 Gdansk, Poland; drjakubwisniewski@gmail.com; 5 Department of Surgery, Faculty of Medicine, Collegium Medicum, University of Rzeszow, 35-959 Rzeszow, Poland; sabinakrupa@o2.pl

**Keywords:** delirium, cardiosurgery, neurosurgery, inflammatory factors

## Abstract

**Background/Objectives**: The increasing number and complexity of cardiac and neurosurgical procedures underscore the importance of effective perioperative care. One of the most serious postoperative complications is delirium, which prolongs hospitalization, increases treatment costs, worsens rehabilitation outcomes, and increases mortality. Identifying biomarkers that predict delirium can improve patient outcomes. The aim of this study was to assess the relationship between C-reactive protein (CRP) levels and the incidence of postoperative delirium in patients after cardiac and neurosurgical procedures hospitalized in the intensive care unit. **Methods:** A retrospective study was conducted on 408 patients (202 undergoing cardiac surgery and 206 undergoing neurosurgery) who underwent surgery between April 2024 to the end of August 2024. Medical records were reviewed for the occurrence of delirium assessed using the Confusion Assessment Method-Intensive Care Unit scale (CAM-ICU), its severity assessed using the Confusion Assessment Method–Intensive Care Unit 7 (CAM-ICU-7), and laboratory test results, with particular emphasis on C-reactive protein levels. CRP levels were measured on postoperative days 1 and 2. **Results:** Postoperative delirium was noted in both groups, more frequently in patients with elevated CRP levels, indicating an active inflammatory process. In the neurosurgical group, episodes of severe delirium occurred primarily after laminectomy, whereas in the cardiac surgery group, they were most common after coronary artery bypass grafting (CABG). **Conclusions:** Elevated CRP levels are associated with a higher risk of postoperative delirium. Monitoring inflammatory parameters and implementing early preventive measures may improve treatment outcomes and shorten hospital stays. Further prospective studies using standardized diagnostic tools are necessary.

## 1. Introduction

Postoperative delirium is a type of delirium occurring in patients after surgical procedures performed under general anesthesia [1,2]. It is a kind of cognitive disorder characterized by sudden, acute and variable impairment of consciousness and attention [3]. Symptoms of postoperative delirium can appear within a short period of time, from 10 min after anesthesia to up to 7 days, and they fluctuate daily [4]. The incidence of postoperative delirium in the general population of patients undergoing surgery ranges from 2.5–3% [5,6]. In patients aged 60–70 years, the incidence is significantly higher, ranging from 10% to 20% [7,8,9]. Urgent surgery carries a 20–45% risk of postoperative mayhem, and thus this risk is 1.5–3 times higher than the same surgery performed as an elective procedure [10,11]. In the case of cardiac or liver surgery, the risk of postoperative mayhem reaches 20–50% [12,13,14].

Postoperative delirium prolongs the patient’s hospital stay by 2–3 days, thereby extending the length of hospitalization in the intensive care unit by 2 days [14,15,16]. Maniar HS. et al. as well as Raasts JW. et al. report that postoperative delirium is also a risk factor for increased 30-day mortality expressed at 7–10%, relative to a 1% risk among patients without delirious elements [16,17]. Additionally, according to Huded CP. et al. and Gleason LJ. et al. postoperative delirium is associated with a deterioration in function, thereby increasing the risk of requiring care for the patient after hospital discharge by 2–3 times [18,19].

The pathogenesis of postoperative mayhem is currently not fully understood. According to many authors, it is a complex and multifactorial process, involving the interaction of multiple predisposing and triggering factors [20,21,22,23]. Modifiable triggers of delirium associated with hospitalization include pain, electrolyte disturbances or hypoxia [23,24,25,26]. Several theories of the pathophysiology of postoperative delirium have been described to date. They are based mainly on the results of studies conducted on animal models [3]. Three basic mechanisms have been identified for the development of postoperative maya, which include inflammation within the nervous system, changes in neurotransmitters, and subclinical vascular events in the brain.

Previous studies have linked elevated perioperative CRP to delirium severity and duration, especially in cardiac surgery and intensive care settings.

Despite this, the literature remains inconsistent, and few comparative analyses exist between different surgical types. Given the high clinical relevance and accessibility of CRP testing, understanding its association with postoperative delirium may provide valuable prognostic insight [27,28].

The primary objective of this study was to assess the association between CRP (C-reactive protein) levels and the incidence and severity of postoperative delirium in patients undergoing cardiac and neurosurgical procedures. A secondary objective was to examine whether the type of surgical procedure modifies this association. The authors hypothesized that elevated C-reactive protein levels in the perioperative and postoperative periods are associated with an increased risk and severity of postoperative delirium after cardiac and neurosurgical procedures.

C-reactive protein was defined in this study as a nonspecific acute-phase protein synthesized by the liver in response to inflammation, primarily via interleukin-6 (IL-6) [29,30,31].

## 2. Materials and Methods

### 2.1. Study Design and Participants

The study was conducted on a group of 408 patients (202 cardiac surgery patients and 206 neurosurgical patients). The study was retrospective, lasting from April 2024 to the end of August 2024. Researchers analyzed the medical records of patients in the study group for delirium and inflammatory parameters. In the first stage of the study, consent was obtained from the Directors of the centers where the study was conducted. A positive opinion from the Bioethics Committee of the Regional Medical Chamber in Gdansk (KB-6/24) was received for the study to begin. After receiving the mentioned approvals, the analysis of the medical records of cardiac and neurosurgical patients was started.

### 2.2. Study Population and Eligibility Criteria

A total of 468 patients were initially screened. Sixty patients were excluded due to incomplete documentation, history of neurological or psychiatric disorders, or missing postoperative CRP measurements. The final study population consisted of 408 patients—202 cardiac surgery patients and 206 neurosurgical patients.

#### 2.2.1. Inclusion Criteria

- Age ≥ 18 years;
- Elective or urgent cardiac or neurosurgical procedure under general anesthesia;
- Availability of perioperative CRP measurements;
- Postoperative delirium assessment using the CAM-ICU scale.

#### 2.2.2. Exclusion Criteria

- Age < 18 years;
- Pre-existing dementia, psychiatric disorders, or addiction to alcohol/psychoactive substances;
- Active infection or fever at admission;
- Chronic inflammatory or autoimmune diseases;
- Active malignancy;
- Missing CRP or CAM-ICU data.

**Figure 1:**
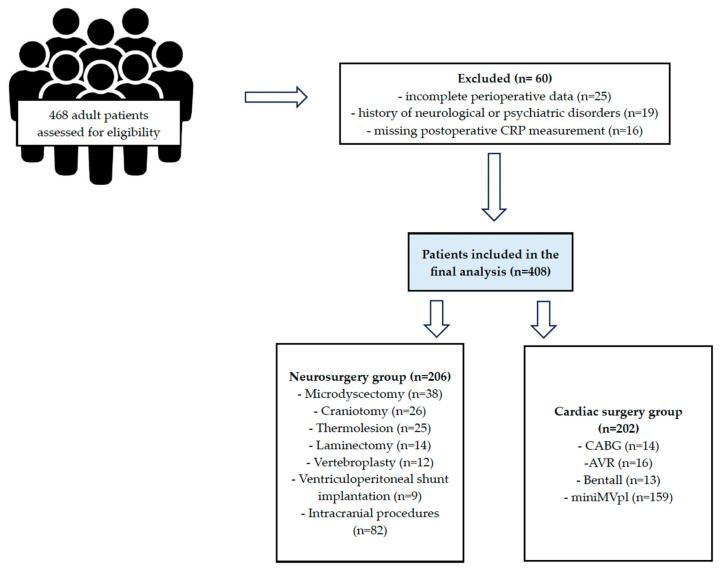
Study Fowchart.

The patient selection process and final allocation are summarized in Figure 1 (Study Flowchart).

### 2.3. Rationale for Selecting Cardiac and Neurosurgical Procedures

Cardiac and neurosurgical procedures were specifically chosen because of their high risk of postoperative delirium and strong links to systemic and neuroinflammatory activation.

Cardiac surgery involving cardiopulmonary bypass triggers systemic inflammatory response, cerebral hypoperfusion, and microembolization—key factors contributing to postoperative delirium. Conversely, neurosurgical interventions directly affect central nervous system integrity and are associated with neuroinflammation, postoperative pain, and sleep–wake disruption.

Comparing these two high-risk surgical groups enabled the evaluation of inflammatory–delirium associations in distinct but pathophysiologically relevant contexts.

### 2.4. Data Collection and Variables

Data were extracted from medical and nursing records, laboratory databases, and perioperative documentation. The collected variables included:

- Demographic data: age, sex;
- Clinical data: type and duration of surgery, length of ICU and total hospital stay;
- Inflammatory marker: perioperative C-reactive protein (CRP) levels;
- Pain assessment: Numeric Rating Scale (NRS);
- Delirium assessment: Confusion Assessment Method for the ICU (CAM-ICU);
- Severity of delirium: Confusion Assessment Method—Intensive Care Unit-7 (CAM-ICU-7).

#### Definitions of Key Variables

- Postoperative delirium was defined as an acute, fluctuating disturbance of attention and cognition diagnosed according to CAM-ICU criteria. Assessments were performed twice daily (8:00 a.m. and 8:00 p.m.) by trained ICU nurses and verified by anesthesiologists. The CAM-ICU assessment includes four main components:
  ♦ Sudden onset and fluctuating course of symptoms, which determines whether there has been a sudden deterioration in mental status or a change in its course over time within the previous 24 h.
  ♦ Inattention—assessed using concentration tests, such as the SAVEAHAART (Sequence of Letters) or gesture responses to verbal stimuli.
  ♦ Disorganized thinking—assessed through a set of simple questions and logical prompts, such as “Does a stone float in water?” or “Count from 1 to 5.”
  ♦ Altered level of consciousness—assessed using the Richmond Agitation-Sedation Scale (RASS); a score other than 0 (i.e., outside the “calm, alert” range) indicates impaired consciousness.

The interpretation of the scale is as follows: delirium is diagnosed when both of the following criteria are present: criterion 1 (sudden onset and fluctuation) and criterion 2 (attention disturbances), and in addition, criterion 3 or 4 (disorganized thinking or altered level of consciousness). This tool demonstrates high sensitivity (94–100%) and specificity (89–95%) for ICU patients [32,33,34].

For grading delirium severity, the CAM-ICU-7 numerical scale was used. This scale converts responses from the CAM-ICU domains into a single numerical score ranging from 0 to 7, where 0–2 = no delirium, 3–5 = mild to moderate delirium, 6–7 = severe delirium [35].

- Pain intensity was assessed using the 11-point Numeric Rating Scale (NRS; 0 = no pain, 10 = worst imaginable pain) every 8 h during the first 48 h after surgery, and whenever patients reported pain.
- C-reactive protein (CRP) concentrations were measured from venous blood sam-ples collected on postoperative day 1 (within 6–8 h after surgery) and on day 2. For analysis, CRP < 5 mg/L was considered normal, 5–10 mg/L indicated develop-ing inflammation, and >10 mg/L active systemic inflammation.

### 2.5. Analysis of Risk Factors

Patient-related factors analyzed for association with postoperative delirium included age, sex, duration of hospitalization, type of surgery, and comorbidities (hypertension, diabetes mellitus, hypercholesterolemia, and active nicotine use). In this cohort, no statistically significant correlation was found between these comorbidities and delirium incidence (*p* > 0.05).

### 2.6. Inflammatory Markers

CRP was the sole inflammatory biomarker included, due to its universal availability, low cost, and reproducibility in perioperative practice. Other markers such as IL-6, TNF-α, and S100B were not routinely measured and thus excluded. Future prospective studies by the authors’ group will aim to incorporate broader neuroinflammatory panels to better elucidate the mechanistic pathways linking systemic inflammation and delirium.

### 2.7. Research Team and Workflow

The research team consisted of five investigators. After receiving institutional and ethical approvals, three researchers analyzed cardiac surgery records and two reviewed neurosurgical cases. Data were cross-validated to ensure reliability and consistency across both subgroups.

### 2.8. Statistical Analysis

Data were analyzed using IBM SPSS Statistics v23.0 (IBM Corp., Armonk, NY, USA).

- The chi-square (χ^2^) test was used to evaluate categorical distributions of inflam-mation (CRP levels) across surgical groups.
- Student’s *t*-test compared mean values between two groups, and one-way ANO-VA (F) was applied for multi-group comparisons.

A *p*-value < 0.05 was considered statistically significant.

### 2.9. Funding Statement

The study was conducted without external funding. The funding bodies had no role in the study design, data collection, statistical analysis, interpretation, or manuscript preparation.

## 3. Results

The study included 408 patients, including 202 cardiac surgery patients (49.51%) and 206 neurosurgical patients (50.49%). In the study group, women accounted for 181 patients and men for 227. The age of cardiac surgery patients ranged from 18 to 78 years (±SD = 13.83), and the age of neurosurgical patients ranged from 18 to 71 years (±SD = 11.82). Patients’ length of stay in the postoperative ward in the cardiac surgery group ranged from 17 to 172 h (±SD = 24.26), and in the neurosurgical group, the maximum was also 172 h (±SD = 27.82). Among cardiac surgery patients, 16 patients (7.92%) underwent AVR (Aortic Valve Replacement), 13 patients (6.44%) underwent Bentall surgery, 159 (78.71) underwent miniMVpl (Mini Mitral Valve Plasty), and 14 patients (6.93%) underwent CABG (Coronary Artery Bypass Grafting). Among neurosurgical patients, lumbar microdiscectomy was performed in 38 patients (18.44%), craniotomy with brain tumor removal in 26 patients (12.62%), and thermolectomy in 25 patients (12.14%). Laminectomy was performed in 14 patients (6.80%), vertebroplasty in 12 patients (5.83%), and ventriculo-peritoneal valve implantation in 9 patients (4.37%). Intracranial procedures, such as endovascular embolization of cerebral aneurysms, were performed in 82 patients (39.81%). Table 1 presents the characteristics of the grouping variables.

A statistically significant difference was found between the patient groups in terms of age (*p* = 0.00001). Patients undergoing cardiac surgery were significantly older, with the majority being over 60 years of age, whereas neurosurgical patients were predominantly between 46 and 60 years old. This result reflects the typical demographic profile of both populations—cardiovascular diseases requiring surgical intervention occur more frequently in elderly patients, while indications for neurosurgical procedures are more common among younger and middle-aged individuals.

### 3.1. C-Reactive Protein Levels by Day and Treatment

We compared the distribution of C-reactive protein for day 1 and day 2 in the cardiac and neurosurgical patient groups. On day 1, the distribution was significantly different (*p* < 0.000001). In the group of cardiac surgery patients, the presence of inflammation (CRP above 10 mg/dL) was not identified. Neurosurgical patients were significantly more likely to have inflammation (CRP above 10 mg/dL) than cardiac surgery patients. On the second day, there were no statistically significant differences (*p* < 0.06) in the distribution of inflammation among the patients studied.

#### 3.1.1. C-Reactive Protein Concentration Level in the Group of Cardiac Surgery Patients Depending on the Procedure

The distribution of inflammation among cardiac surgery patients varied significantly depending on the type of procedure, both on the first and second postoperative days. On the first day, the highest incidence of inflammation was observed after CABG surgery (70.79%), followed by markedly lower rates after AVR (7.92%), Bentall (6.44%), and miniMVpl (2.97%) procedures.

On the second postoperative day, inflammation remained most frequent in patients after CABG (58.91%), while considerably lower rates were recorded after miniMVpl (6.93%), Bentall (5.45%), and AVR (4.95%) procedures.

#### 3.1.2. C-Reactive Protein Concentration Level in the Group of Neurosurgical Patients Depending on the Procedure

The distribution of inflammation in the neurosurgical patient group, depending on the procedure performed, was significantly different on the first day (*p* < 0.00001). Inflammation was found in patients with laminectomy (6.80%) and microdiscectomy (0.49%). In contrast, no inflammation was observed on the second day for any procedure, and the distribution of CRP levels normalized, with similar levels between procedures (*p* = 0.73). The results are shown in Table 2.

### 3.2. Incidence of Delirium in Different Groups of Patients and Different Treatments on the First and Second Day of Hospitalization

In the study group of patients, the degree of delirium ranged from level 0—no delirium to level 7—severe delirium. The average level of delirium in the group of cardiac surgery patients was significantly higher than in the group of neurosurgical patients on day 1 and day 2. Table 3 presents detailed data.

We compared the incidence of delirium on day 1 and day 2 in the cardiac and neurosurgical patient groups. On day 1 and day 2, the distribution was significantly different (*p* < 0.000001). In the group of cardiac surgery patients, we identified a higher incidence of mild-moderate delirium and more frequent cases of severe delirium on both day 1 and day 2 compared to neurosurgical patients. The results are shown in Table 4.

#### 3.2.1. Distribution of the Incidence of Delirium in Cardiac Surgery Patients According to the Type of Surgery

The distribution of the incidence of delirium in cardiac surgery patients, depending on the procedure performed, was significantly different on the first day. The highest number of situations of development of severe delirium on the first day was shown for the CABG procedure (7.43%). Next, less than 4% of severe delirium on day 1 was observed with AVR surgery (3.96%), while after Bentall surgery, a severe episode of delirium occurred in 3.96% of the subjects. There was no incidence of severe delirium in the miniMVpl procedure.

On the second day, there were no statistically significant (*p* = 0.07) differences in the incidence of delirium between treatments among cardiac surgery patients. The results of the study are shown in Table 5.

#### 3.2.2. Distribution of the Incidence of Delirium in Neurosurgical Patients According to the Type of Surgery

The incidence of delirium among neurosurgical patients varied significantly on the first postoperative day depending on the type of procedure performed. The highest rate of severe delirium on day 1 was observed following laminectomy, whereas markedly fewer cases were noted after lumbar microdiscectomy.

On the second postoperative day, no statistically significant differences in the incidence of delirium were observed between the analyzed neurosurgical procedures. Detailed results are presented in Table 6.

### 3.3. Correlation Between C-Reactive Protein Levels and the Occurrence and Severity of Delirium

To assess the relationship between C-reactive protein (CRP) levels and the incidence of delirium, a one-way ANOVA (F test) was conducted to compare mean delirium scores between patients with elevated and normal CRP levels on postoperative days 1 and 2. Statistically significant differences were observed on day 1 in both the cardiac and neurosurgical groups, whereas no significant associations were found on day 2.

On day 1, cardiac surgery patients with elevated CRP levels exhibited significantly higher delirium scores compared to those with normal CRP concentrations. A similar pattern was observed in the neurosurgical group, where patients with elevated CRP also demonstrated significantly higher delirium scores.

Additionally, significant intergroup differences were identified. Cardiac surgery patients had markedly higher delirium scores than neurosurgical patients on both postoperative days 1 and 2. Detailed results are presented in Table 7.

#### 3.3.1. Occurrence of Delirium in Cardiac Surgery Patients Depending on the Type of Surgery and CRP Level

In the cardiac surgery group, statistically significant differences in the severity of delirium were observed between the types of procedures performed. Patients undergoing aortic valve replacement (AVR) and Bentall procedures exhibited markedly higher delirium intensity compared with those who underwent coronary artery bypass grafting (CABG) or minimally invasive mitral valve plasty (miniMVpl).

However, no statistically significant differences in delirium severity were identified between patients with normal CRP levels and those with elevated CRP indicating inflammation, either on the first or second postoperative day within the respective procedure subgroups. Detailed results are presented in Table 8.

#### 3.3.2. Occurrence of Delirium in Neurosurgical Patients Depending on the Type of Surgery and CRP Level

In the neurosurgical patient group, statistically significant differences in delirium severity were observed between the types of procedures on both the first and second postoperative days. Laminectomy was associated with higher postoperative C-reactive protein (CRP) levels, which coincided with greater delirium intensity.

However, no statistically significant differences in delirium severity were identified between patients with normal and elevated CRP levels on either postoperative day, regardless of the specific surgical procedure performed. Detailed results are presented in Table 9.

### 3.4. Correlation Between NRS, Type of Surgical Procedures and Delirium

As part of the present study, the authors additionally assessed the intensity of postoperative pain using the Numeric Rating Scale (NRS) and analyzed its potential impact on the occurrence and severity of postoperative delirium. The aim of this supplementary assessment was to determine whether the intensity of perceived pain could serve as a contributing factor to the development of postoperative cognitive disturbances.

The relationship between NRS values and the incidence of delirium was analyzed across different types of cardiac and neurosurgical procedures to evaluate whether inadequate pain control or greater pain intensity—resulting from the extent and invasiveness of surgery—could affect cognitive functioning in the early postoperative period.

The analysis revealed a clear association between the type of surgical procedure and the level of postoperative pain in both study groups—cardiac and neurosurgical—which, in turn, positively correlated with the occurrence of delirium. These findings indicate an indirect but significant influence of pain intensity on the development of postoperative cognitive disturbances.

Procedures of greater extent and longer duration were associated with a stronger inflammatory response, higher NRS scores, and an increased risk of delirium. The results emphasize the importance of effective, multimodal postoperative analgesia and the need for close monitoring of patients with a high risk of developing delirium.

Detailed results of the analysis are presented in Table 10 and Table 11.

## 4. Discussion

Postoperative delirium is an acute neurocognitive disorder characterized by sudden changes in mental state, affecting cognitive functions, consciousness, and alertness [36]. In this study, delirium severity was quantified using the CAM-ICU-7 scale, which enables standardized scoring of delirium intensity on a 0–7 scale, thus ensuring methodological consistency between diagnostic assessment and statistical analysis. As reported by Plaschke K. et al. and Hirsch J. et al., in the immediate postoperative period, there is an increased level of systemic inflammatory mediators, which remain high even in the days following surgery [37,38]. Liu X et al. [30] report, on the other hand, that a postoperative increase in C-reactive protein concentration is associated with an increased risk of postoperative delirium. Importantly, the authors of this report also observed that a preoperative increase in C-reactive protein concentration is an independent predictor of postoperative delirium development. This confirms the hypothesis of an increased risk of developing postoperative delirium secondary to preoperative pathologies. The research conducted by the authors of this manuscript is consistent with previously published studies, thereby confirming, among other things, the results of the observations by Liu X et al. [30].

Studies conducted by Hu N. et al., Yang S. et al., and Cao Y et al., showed that the inflammatory process occurring in the periphery of the body can cause the loss of structural and functional integrity of the blood–brain barrier, thereby enabling the migration of inflammatory mediators and inflammatory cells to the central nervous system [39,40,41,42]. The accumulation of inflammatory factors in the nervous tissue of the brain leads to a loss of synaptic plasticity, neuroapoptosis, and impaired neurogenesis. This, in turn, constitutes a risk factor for the development of delirium, including postoperative delirium [43,44,45]. In our study, the incidence and severity of postoperative delirium differed significantly between cardiac and neurosurgical patients. Delirium occurred most frequently after CABG and laminectomy procedures. Both operations are characterized by strong systemic and neuroinflammatory activation. In cardiac surgery, the use of cardiopulmonary bypass, transient cerebral hypoperfusion, and microembolization may contribute to postoperative cognitive dysfunction, consistent with previous findings by Bruggemans, Zhuang et al., and Stanley et al. [46,47,48]. In turn, in neurosurgical patients, particularly those undergoing laminectomy, delirium may be promoted by intense postoperative pain, high-dose opioid use, sleep–wake disturbances, and limited mobility during recovery. These factors amplify stress-related catecholamine release and systemic inflammation, thereby triggering neurochemical pathways associated with acute confusion. Adam EH. et al. in a recent analysis proved that acetylcholine activity in patients with postoperative delirium both before cardiac surgery and 2 days after it is reduced and is an independent risk factor for the development of delirium [49]. Similarly, Zhao B. et al. confirmed in their study the observation of reduced acetylcholinesterase activity in a group of elderly patients undergoing non-cardiac surgery [50]. Guo Y. et al. and Oliveira ER. et al. noted that diseases such as hypertension, atrial fibrillation, and heart valve diseases, which pose a risk of vascular events, are an independent factor in the development of postoperative delirium episodes [51,52]. This supports the interpretation that vascular burden and systemic inflammation act synergistically in precipitating delirium after major cardiovascular procedures.

Although, according to the work of Mrkobrad M. et al. and a prospective cohort study published in The Lancet on occult perioperative stroke among patients undergoing non-cardiac surgery, radiological signs of cerebral ischemia can be observed in 7–10% of elderly patients, which is associated with a twofold increase in the risk of postoperative delirium [53,54]. Hori D. et al. conducted a retrospective study analyzing the relationship between cerebral perfusion pressure and delirium. They found that cerebral perfusion pressure above the autoregulation threshold is an independent factor in the development of postoperative delirium [55].

The observed association between elevated CRP and delirium in our cohort reinforces the role of systemic inflammation as a key mechanism. Elevated CRP reflects the intensity of the inflammatory response and is linked to cytokine release, endothelial dysfunction, and blood–brain barrier permeability, facilitating neuroinflammation and neuronal dysregulation [28,29,56,57]. The relationship between CRP and delirium severity suggests that biochemical markers of inflammation may serve as early warning indicators of postoperative neurocognitive complications.

Finally, although our analysis demonstrates the influence of the surgical procedure on delirium occurrence, this factor should be interpreted within a broader context. Delirium is a multifactorial syndrome influenced by patient-related (age, comorbidities, baseline cognition), surgical (procedure type, invasiveness, blood loss), and perioperative factors (anesthesia, analgesia, pain, and sleep disruption). Thus, the type of surgery likely reflects the cumulative physiological and inflammatory burden rather than an isolated determinant of delirium.

Although the current study demonstrated highly significant statistical associations between postoperative C-reactive protein (CRP) levels and delirium incidence, the analysis remains observational. It lacks multivariate adjustment for potential perioperative confounders. Several variables—such as anesthetic technique, sedative exposure (e.g., benzodiazepines, propofol), intraoperative hypotension, opioid use, and duration of mechanical ventilation—may simultaneously influence systemic inflammatory response and delirium risk [29,31,36]. The absence of these covariates in a multivariable model limits causal inference. Moreover, CRP, while a sensitive marker of systemic inflammation, is non-specific and can rise in response to global surgical stress, tissue injury, or infection, rather than mechanisms directly related to neuroinflammation and acute cognitive dysfunction [27,28]. In addition, the lack of reported effect sizes (e.g., odds ratios, β-coefficients) and confidence intervals precludes assessment of the clinical magnitude and precision of the observed associations. Therefore, the present results should be regarded as exploratory, supporting the hypothesis that systemic inflammation contributes to the pathogenesis of postoperative delirium but requiring confirmation in future prospective, multicenter studies with comprehensive adjustment for perioperative variables and inclusion of broader biomarker panels (e.g., CRP, IL-6, TNF-α, S100B, cholinesterase) to determine their true predictive value for postoperative neurocognitive disorders.

## 5. Study Limitation

This study has several limitations that should be acknowledged.

- **First,** its retrospective and single-center design limits the ability to establish causal relationships between C-reactive protein (CRP) levels and the development of postoperative delirium. Although strict inclusion and exclusion criteria were applied, the retrospective nature of the analysis may have resulted in incomplete or non-uniform documentation of perioperative variables, including pain intensity, anesthetic agents, and sedative use.
- **Second**, delirium assessment was based solely on the Confusion Assessment Method for the Intensive Care Unit (CAM-ICU) scale. While this tool has high diagnostic accuracy, it does not capture the full spectrum of cognitive fluctuations and subtle neurobehavioral changes that may occur postoperatively, potentially leading to underestimation of mild cases.
- **Third**, CRP was the only inflammatory marker analyzed. The study did not include other relevant biomarkers of systemic or neuroinflammation (e.g., interleukin-6, TNF-α, or S100B protein), which could have provided a broader mechanistic understanding of the inflammatory contribution to delirium.
- **Fourth**, perioperative factors such as the depth of anesthesia, intraoperative hemodynamic fluctuations, duration of surgery, or postoperative pharmacologic management were not systematically controlled or included in the statistical model, though these factors are known to influence delirium risk.
- **Finally**, the study population consisted exclusively of patients from a single tertiary hospital, which may limit the generalizability of the results to other surgical populations and healthcare settings.

Future multicenter, prospective studies incorporating continuous monitoring of inflammatory markers and standardized cognitive assessments are warranted to validate these findings and better elucidate the causal mechanisms linking inflammation and postoperative delirium.

## 6. Conclusions

This retrospective study demonstrated that elevated C-reactive protein (CRP) levels are significantly associated with a higher incidence and severity of postoperative delirium in patients undergoing cardiac and neurosurgical procedures. The relationship was particularly evident on the first postoperative day, with CABG in the cardiac surgery group and laminectomy in the neurosurgical group being most strongly linked to severe delirium episodes. These observations indicate that both surgical stress and inflammatory activation contribute to postoperative neurocognitive dysfunction.

Our findings emphasize that delirium development is multifactorial—driven by patient age, type and invasiveness of surgery, and the magnitude of the inflammatory response. Routine monitoring of CRP in the early perioperative period may provide a simple and accessible tool for identifying patients at increased risk of delirium, allowing timely preventive and therapeutic interventions.

However, given the retrospective and single-center design of this study, the results should be interpreted with caution. Future large-scale, prospective, and multicenter studies using standardized delirium assessment tools are warranted to confirm the predictive value of CRP and to explore its integration into comprehensive perioperative risk stratification models.

## Figures and Tables

**Table 1:** Characteristics of grouping variables.

Type	Category	Cardiac Surgical Patients		Neurosurgical Patients		χ^2^	*p*
		N	%	N	%		
Gender	Female	90	44.55%	91	44.17%	0.01	0.93
	Male	112	55.45%	115	55.83%		
Age group	<30	17	8.42%	18	8.74%	26.57	0.00001
	31–45	40	19.80%	47	22.82%		
	46–60	74	36.63%	112	54.37%		
	>60	71	35.15%	29	14.08%		
Duration of stay	0–24	69	34.16%	76	36.89%	3.03	0.21
	25–48	103	50.99%	89	43.20%		
	above 48	30	14.85%	41	19.90%		
**Surgical procedures**							
Patient Type	Type of treatment					N	%
Cardiac Surgical Patients	AVR (aortic valve replacement)					16	7.92%
	Bentall					13	6.44%
	miniMVpl (mini mitral valve plasty)					159	78.71%
	CABG (coronary artery bypass grafting)					14	6.93%
Neurosurgical patients	Microdiscectomy of the lumbar spine					38	18.44%
	Craniotomy with removal of a brain tumor					26	12.62%
	Intracranial procedures					82	39.81%
	Thermolesion					25	12.14%
	Laminectomy					14	6.80%
	Vertebroplasty					12	5.83%
	Ventriculoperitoneal shunt implantation					9	4.37%

**Table 2:** Significance of the distribution of C-reactive protein concentration levels on the 1st and 2nd day of hospitalization, depending on the procedure in neurosurgery patients.

Neurosurgical Patients								
CRP	Norm		Development of Inflammation		Inflammation		χ^2^	*p*
	N	%	N	%	N	%		
CRP 1st day								
Microdiscectomy of the lumbar spine (MLD)	20	9.71%	17	8.25%	1	0.49%	185.25	0.00001
Craniotomy with removal of a brain tumor (C&R)	6	2.91%	20	9.71%	0	0%		
Intracranial procedures (IP)	3	1.46%	79	38.35%	0	0%		
Thermolesion	3	1.46%	22	10.68%	0	0%		
Laminectomy	0	0%	0	0%	14	6.80%		
Vertebroplasty	3	1.46%	9	4.37%	0	0%		
Ventriculoperitoneal shunt implantation (VP)	3	1.46%	6	2.91%	0	0%		
CRP 2nd day								
Microdiscectomy of the lumbar spine (MLD)	5	2.43%	33	16.02%	0	0%	4.35	0.73
Craniotomy with removal of a brain tumor (C&R)	7	3.40%	19	9.22%	0	0%		
Intracranial procedures (IP)	15	7.28%	67	32.52%	0	0%		
Thermolesion	2	0.97%	23	11.17%	0	0%		
Laminectomy	2	0.97%	12	5.83%	0	0%		
Vertebroplasty	2	0.97%	10	4.85%	0	0%		
Ventriculoperitoneal shunt implantation (VP)	1	0.49%	8	3.88%	0	0%		

*p*—significance level; χ^2^—chi-square test; N—number; %—percentage; MLD—micro-lumbar discectomy; C&R—craniotomy and resection; IP—intracranial procedures; VP—ventriculoperitoneal shunt.

**Table 3:** Comparison of the occurrence of delirium levels in patient groups on individual days.

Delirium	Cardiosurgery Patients					Neurosurgery Patients					F	*p*
	N	$\bar{x}$	Min	Max	SD	N	$\bar{x}$	Min	Max	SD		
1st day	202	1.62	0	7	2.35	206	1.07	0	7	1.59	7.86	0.005
2nd day	202	1.15	0	7	1.62	206	0.52	0	7	0.74	25.63	0.000001

N—number; X—weighted mean; Min—minimum, Max—maximum, SD—standard deviation; F—ANOVA analysis test, *p*—significance level.

**Table 4:** Occurrence of delirium levels in individual patient groups.

Delirium	Cardiosurgery Patients						Neurosurgery Patients						χ^2^	*p*
	No Delirium		Mild-Moderate Delirium		Severe Delirium		No Delirium		Mild-Moderate Delirium		Severe Delirium			
	N	%	N	%	N	%	N	%	N	%	N	%		
1st day	154	76.24%	17	8.42%	31	15%	192	93.20%	1	0.49%	13	6.31%	28.19	<0.000001
2nd day	174	86.14%	18	8.91%	10	5%	204	99.03%	1	0.49%	1	0.49%	28.38	<0.000001

N—number; %—percentage; χ^2^ chi-square test; *p*—significance level.

**Table 5:** The occurrence of delirium in a group of cardiac surgery patients depends on the type of procedure performed.

Cardiosurgery Patients								
Delirium	No Delirium		Mild-Moderate Delirium		Severe Delirium		χ^2^	*p*
	N	%	N	%	N	%		
1st day								
AVR	6	3%	2	0.99%	8	3.96%	42.01	<0.000001
Bentall	3	1%	2	0.99%	8	3.96%		
CABG	131	64.85%	13	6.44%	15	7.43%		
miniMVpl	14	6.93%	0	0%	0	0%		
2nd day								
AVR	13	6%	3	1.49%	0	0%	11.32	0.07
Bentall	11	5%	0	0.00%	2	1%		
CABG	136	67.33%	15	7.43%	8	4%		
miniMVpl	14	7%	0	0%	0	0%		

N—number; %—percentage; χ^2^—chi-square test; *p*—significance level.

**Table 6:** The occurrence of delirium in a group of neurosurgical patients depends on the type of procedure performed.

Neurosurgery Patients								
Delirium	No Delirium		Mild-Moderate Delirium		Severe Delirium		χ^2^	*p*
	N	%	N	%	N	%		
1st day								
Microdiscectomy of the lumbar spine	36	17.48%	0	0%	1	0.49%	87.42	<0.000001
Craniotomy with removal of a brain tumor	26	12.62%	0	0%	0	0%		
Intracranial procedures	82	39.81%	0	0%	0	0%		
Thermolesion	25	12.14%	0	0%	0	0%		
Laminectomy	1	0.49%	1	0.49%	12	5.83%		
Vertebroplasty	12	5.83%	0	0%	0	0%		
Ventriculoperitoneal shunt implantation	9	4.37%	0	0%	0	0%		
2nd day								
Microdiscectomy of the lumbar spine	38	18.44%	0	0%	0	0%	11.05	0.68
Craniotomy with removal of a brain tumor	28	13.59%	0	0%	0	0%		
Intracranial procedures	82	39.81%	0	0%	0	0%		
Thermolesion	25	12.14%	0	0%	0	0%		
Laminectomy	12	5.83%	1	0.49%	1	0.49%		
Vertebroplasty	12	5.83%	0	0%	0	0%		
Ventriculoperitoneal shunt implantation	9	4.37%	0	0%	0	0%		

N—number; %—percentage; χ^2^—chi-square test; *p*—significance level.

**Table 7:** Comparison of mean delirium levels depending on CRP levels in cardiac and neurosurgical patient groups.

CRP/Delirium	Cardiosurgery Patients		Neurosurgery Patients		*t*	*p*
	$\bar{x}$	SD	$\bar{x}$	SD		
1st day						
Norm	0.58	1.06	0.82	1.06		
Development of inflammation	1.76	2.44	0.69	0.73		
Inflammation			5.60	2.16		
Total	1.62	2.35	1.07	1.59	−2.80	0.01
**F**	5.47		179.43			
***p***	0.02		<0.000001			
2nd day						
Norm	0.83	0.72	0.59	0.50		
Development of inflammation	1.25	1.80	0.51	0.78		
Total	1.15	1.62	0.52	0.74	−5.06	<0.000001
**F**	2.48		0.30			
***p***	0.12		0.58			

$\bar{x}$—weighted mean; SD—standard deviation; *t*—Student’s test; *p*—significance level; F—ANOVA analysis test.

**Table 8:** Comparison of mean delirium levels depending on CRP levels in groups of individual cardiac surgery procedures.

CRP/Delirium	Cardiosurgery Patients								F	*p*
	AVR		Bentall		CABG		miniMVpl			
	$\bar{x}$	SD	$\bar{x}$	SD	$\bar{x}$	SD	$\bar{x}$	SD		
1st day										
Norm					0.63	1.26	0.50	0.53		
Development of inflammation	4.06	2.93	4.85	2.48	1.29	2.05	0.33	0.52		
Total	4.06	2.93	4.85	2.48	1.22	1.99	0.43	0.51	21.92	<0.000001
F					1.60		0.34			
*p*					0.21		0.57			
2nd day										
Norm	1.00	1.55	0.50	0.71	0.83	0.55				
Development of inflammation	0.50	0.97	1.55	2.50	1.38	1.85	0.50	0.52		
Total	0.69	1.20	1.38	2.33	1.24	1.64	0.50	0.52	1.45	0.23
F	0.64		0.32		3.46					
*p*	0.44		0.58		0.06					

$\bar{x}$—weighted average; SD—standard deviation; *p*—significance level; F—ANOVA test.

**Table 9:** Comparison of mean delirium levels depending on CRP levels in groups of individual neurosurgical procedures.

CRP/Delirium	Neurosurgical Patients														F	*p*
	Microdiscectomy of the Lumbar Spine		Craniotomy with Removal of a Brain Tumor		Intracranial Procedures		Ventriculoperitoneal Shunt Implantation		Thermolesion		Laminectomy		Vertebroplasty			
	$\bar{x}$	SD	$\bar{x}$	SD	$\bar{x}$	SD	$\bar{x}$	SD	$\bar{x}$	SD	$\bar{x}$	SD	$\bar{x}$	SD		
CRP 1st day																
Norm	1.11	1.37	0.67	0.52	0.33	0.58	0.67	0.58	0.33	0.58	0.00		0.67	0.58	71.01	<0.000001
Development of inflammation	0.71	0.69	0.35	0.59	0.67	0.71	0.83	0.98	1.05	0.79	0.00		0.56	0.73		
Inflammation	0.00										6.00	1.56				
Total	1.74	2.05	0.42	0.58	0.66	0.71	0.78	0.83	0.96	0.79	6.00	1.56	0.58	0.67		
F	0.95		1.41		0.66		0.07		2.26				0.06			
*p*	1.72		0.25		0.42		0.80		0.15				0.82			
CRP 2nd day																
Norm	0.83	2.00	0.43	0.53	0.60	0.51	1.00		0.50	0.71			1.00	0.00	4.71	<0.000001
Development of inflammation	0.72	0.96	0.42	0.51	0.49	0.50	0.63	0.52	0.30	0.47			0.40	0.52		
Total	0.73	0.96	0.42	0.50	0.51	0.50	0.67	0.50	0.32	0.48	1.50	1.99	0.50	0.52		
F	0.09		0.00		0.56		0.47		0.30				2.50			
*p*	1.72		0.97		0.46		0.52		0.59				0.14			

$\bar{x}$—weighted average; SD—standard deviation; *p*—significance level; F—ANOVA test.

**Table 10:** Correlation between NRS, type of cardio surgery procedures and delirium.

Cardio Surgery Patients				
Procedures	NRS		Delirium	
	$\bar{x}$	SD	$\bar{x}$	SD
1st day				
AVR	5.29	1.02	1.22	1.99
Bentall	6.73	0.92	4.06	2.93
CABG	2.98	0.34	0.43	0.51
miniMVpl	6.49	1.01	4.85	2.48
F	44.05		21.92	
*p*	<0.001		<0.003	
2nd day				
AVR	4.62	1.06	1.24	1.64
Bentall	4.27	1.04	0.69	1.20
CABG	2.98	0.34	0.50	0.52
miniMVpl	5.19	1.50	1.38	2.33
F	12.43		1.45	
*p*	<0.002		0.23	

$\bar{x}$—weighted average; SD—standard deviation; *p*—significance level; F—ANOVA test.

**Table 11:** Correlation between neurosurgical procedure, NRS and delirium.

Neurosurgery Patients				
Procedures	NRS		Delirium	
	$\bar{x}$	SD	$\bar{x}$	SD
1st day				
Laminectomy	0.99	0.41	0.96	0.79
Craniotomy with removal of a brain tumor	5.19	1.12	0.63	0.68
Intracranial procedures	2.20	0.15	1.11	1.37
Microdiscectomy of the lumbar spine	5.58	1.26	0.66	0.71
Thermolesion	4.88	1.07	0.58	0.67
Removal of an intraspinal tumor	6.42	1.76	0.42	0.58
Vertebroplasty	4.75	1.32	0.78	0.83
Ventriculoperitoneal shunt implantation	6.16	1.47	6.00	1.57
F	62.58		71.01	
*p*	<0.000001		<0.000001	
2nd day				
Laminectomy	4.42	1.32	0.32	0.48
Craniotomy with removal of a brain tumor	4.78	1.48	0.26	0.45
Intracranial procedures	5.17	1.58	0.47	0.51
Microdiscectomy of the lumbar spine	4.71	1.47	0.51	0.50
Thermolesion	4.44	1.00	0.50	0.52
Removal of an intraspinal tumor	4.32	1.37	0.42	0.50
Vertebroplasty	4.14	1.64	0.67	0.50
Ventriculoperitoneal shunt implantation	4.47	0.92	1.50	1.99
F	0.94		4.71	
*p*	0.48		0.0001	

$\bar{x}$—weighted average; SD—standard deviation; *p*—significance level; F—ANOVA test.

## Data Availability

The original contributions presented in this study are included in the article. Further inquiries can be directed to the corresponding author.

## Abbreviations

The following abbreviations are used in this manuscript:

CUM-ICU	Confusion Assessment Method-Intensive Care Unit
CABG	Coronary Artery Bypass Grafting
CRP	C-reactive protein
miniMVpl	Mini Mitral Valve Plasty
AVR	Aortic Valve Replacement
ICU	Intensive Care Unit
NRS	Numeric Rating Scale
